# Association between *METTL3* gene polymorphisms and neuroblastoma susceptibility: A nine‐centre case‐control study

**DOI:** 10.1111/jcmm.15576

**Published:** 2020-07-02

**Authors:** Jun Bian, Zhenjian Zhuo, Jinhong Zhu, Zhonghua Yang, Zhang Jiao, Yong Li, Jiwen Cheng, Haixia Zhou, Suhong Li, Li Li, Jing He, Yanfei Liu

**Affiliations:** ^1^ Department of General Surgery Xi'an Children’s Hospital Xi'an Jiaotong University Affiliated Children's Hospital Xi'an China; ^2^ Department of Pediatric Surgery Guangzhou Institute of Pediatrics Guangdong Provincial Key Laboratory of Research in Structural Birth Defect Disease Guangzhou Women and Children’s Medical Center Guangzhou Medical University Guangzhou China; ^3^ Department of Clinical Laboratory Biobank, Harbin Medical University Cancer Hospital Harbin China; ^4^ Department of Pediatric Surgery Shengjing Hospital of China Medical University Shenyang China; ^5^ Department of Pediatric Surgery The First Affiliated Hospital of Zhengzhou University Zhengzhou China; ^6^ Department of Pediatric Surgery Hunan Children’s Hospital Changsha China; ^7^ Department of Pediatric Surgery The Second Affiliated Hospital of Xi’an Jiaotong University Xi’an China; ^8^ Department of Hematology The Second Affiliated Hospital and Yuying Children’s Hospital of Wenzhou Medical University Wenzhou China; ^9^ Department of Pathology Children Hospital and Women Health Center of Shanxi Taiyuan China; ^10^ Kunming Key Laboratory of Children Infection and Immunity, Yunnan Key Laboratory of Children’s Major Disease Research Yunnan Institute of Pediatrics Research Yunnan Medical Center for Pediatric Diseases Kunming Children’s Hospital Kunming China

**Keywords:** case‐control study, *METTL3*, neuroblastoma, polymorphism, risk

## Abstract

Neuroblastoma ranks as the most commonly seen and deadly solid tumour in infancy. The aberrant activity of m^6^A‐RNA methyltransferase METTL3 is involved in human cancers. Therefore, functional genetic variants in the *METTL3* gene may contribute to neuroblastoma risk. In the current nine‐centre case‐control study, we aimed to analyse the association between the *METTL3* gene single nucleotide polymorphisms (SNPs) and neuroblastoma susceptibility. We genotyped four *METTL3* gene SNPs (rs1061026 T>G, rs1061027 C>A, rs1139130 A>G, and rs1263801 G>C) in 968 neuroblastoma patients and 1814 controls in China. We found significant associations between these SNPs and neuroblastoma risk in neither single‐locus nor combined analyses. Interestingly, in the stratified analysis, we observed a significant risk association with rs1061027 AA in subgroups of children ≤ 18 months of age (adjusted OR = 1.87, 95% CI = 1.03‐3.41, *P* = .040) and females (adjusted OR = 1.86, 95% CI = 1.07‐3.24, *P* = .028). Overall, we identified a significant association between *METTL3* gene rs1061027 C>A polymorphism and neuroblastoma risk in children ≤18 months of age and females. Our findings provide novel insights into the genetic determinants of neuroblastoma.

## 1 INTRODUCTION

Neuroblastoma is a solid childhood cancer arising from sympatho‐adrenergic neuronal progenitors.^1^ It accounts for approximately 5% of all paediatric cancers, but disproportionally causes 12% cancer mortality in children.^2^ The incidence rate of neuroblastoma in America is nearly 1 out of 7000.^3^ Yet, the incidence rate of neuroblastoma in China is about 1 out of 13 000.^4^ Unlike other paediatric malignancies, neuroblastoma is characterized by high phenotypic heterogeneity.^5^ Clinical outcomes of cases vary significantly from spontaneous recovery without treatment to therapy‐resistant progression.^6^, ^7^ Survival rate could be achieved at least 95% in patients with a non–high‐risk (low‐ and intermediate‐risk) neuroblastoma.^8^ Conversely, only 50% of patients with high‐risk neuroblastoma achieve long‐term survival.^9^, ^10^

Over the past decades, significant advances have been made towards understanding the determinants of neuroblastoma risk.^11^, ^12^ Environmental or parental exposures were reported to predispose to neuroblastoma, but warrant more validations.^13^, ^14^ Previous research has suggested that there is a strong genetic component underlying neuroblastoma susceptibility.^15^ For example, most of the familial neuroblastoma harboured mutations in genes *ALK* ^16^, ^17^ and *PHOX2B*.^18^ A clinical trial of an inhibitor of *ALK* was launched soon after the initial discovery of *ALK* mutations. Moreover, researchers have unceasingly found predisposing genetic polymorphisms in sporadic neuroblastoma.^19^, ^20^, ^21^, ^22^, ^23^, ^24^, ^25^, ^26^ To be noted, all the identified genetic variations so far only revealed a small part of the genetic landscape of this malignancy. Therefore, it would be of translational interest to determine more causal genetic risk variants for improving the prevention and prognosis of neuroblastoma.

N6‐methyladenosine (m^6^A) is the most prevalent modification in RNA, especially mRNA.^27^, ^28^ The m^6^A modification mainly regulates gene expression at the post‐transcriptional levels by affecting mRNA stability, mRNA translation and splicing.^23^ m^6^A modifications are installed by RNA methyltransferases (METTL3, METTL14 and WTAP, known as ‘writers’), removed by the demethylases (FTO and ALKBH5, known as ‘erasers’), and recognized by m^6^A‐binding proteins (YTHDF1/2/3 and IGF2BP1, known as ‘readers’).^29^, ^30^, ^31^, ^32^ Emerging evidence suggests that dysregulated m^6^A modification is tightly implicated in various diseases, especially cancers.^33^, ^34^, ^35^ Several lines of evidence have pointed to the involvement of METTL3 in the development and progression of several types of cancer. However, studies on *METTL3* gene single nucleotide polymorphisms (SNPs) and cancer risk are very scarce.

To identify *METTL3* genetic variations that confer susceptibility to neuroblastoma, we performed this multi‐centre epidemiology study.

## 2 MATERIALS AND METHODS

### 2.1 Sample selection

The current study is a hospital‐based case‐control study of neuroblastoma with participants recruited from nine hospitals in China (Guangzhou, Zhengzhou, Wenzhou, Xi'an*2, Taiyuan, Kunming, Changsha, Shenyang). Subjects were patients who were diagnosed and histologically confirmed with neuroblastoma. All specimens were obtained at the time of diagnosis and annotated with clinical information, including gender, age at diagnosis, site of origin, and INSS disease stage. Eligibility criteria for control subjects were as follows: (a) self‐reporting as Chinese; (b) written consent to donate 1.5 mL peripheral blood; (c) no other medical disorders, including cancer; and (d) frequency matched to cases on age and gender. The Institutional Review Board of Guangzhou Women and Children's Medical Center approved the study, and written informed consent was obtained from all subjects by nursing staff under the direction of clinicians. Details are provided in previous publications.^36^, ^37^

### 2.2 Polymorphism selection and genotyping

Potentially functional SNPs in the *METTL3* gene were screened out from the dbSNP database and SNPinfo software.^38^, ^39^ In brief, we searched for potentially functional candidate SNPs located in the 5′‐flanking region, 5′‐untranslated region, 3′‐untranslated region and exon of *METTL3*. Moreover, the included SNPs should conform to: (a) the minor allele frequency >5% for Chinese Han subjects; (b) putative functional potential SNPs, which might affect transcription activity or binding capacity of the microRNA‐binding site; and (c) SNPs in low linkage disequilibrium with each other (*R* ^2^ > .8). Following these criteria, four SNPs (rs1061026 T>G, rs1061027 C>A, rs1139130 A>G, and rs1263801 G>C) in the *METTL3* gene were selected for the Chinese sample genotyping. As shown in Figure S1, there was no significant LD (*R* ^2^ < .8) among these four SNPs of *METTL3* (*R* ^2^ = .036 between rs1061026 and rs1061027, *R* ^2^ = .009 between rs1061026 and rs1139130, *R* ^2^ = .248 between rs1061026 and rs1263801, *R* ^2^ = .453 between rs1061027 and rs1139130, *R* ^2^ = .459 between rs1061027 and rs1263801, *R* ^2^ = .387 between rs1139130 and rs1263801). Blood samples were stored at −80°C. DNA was extracted according to standard procedure, followed by genotyping using TaqMan methodology.^40^, ^41^, ^42^ Laboratory personnel were blinded to case/control status. We also repeatedly genotyped 10% randomly selected sample to assess the genotyping error rate and obtained concordance rates of 100%.

### 2.3 Statistical analysis

Compliance of alleles at individual loci with the Hardy‐Weinberg equilibrium (HWE) was measured in controls using a chi‐square test. Differences in selected demographic variables between cases and controls were assessed by the chi‐square test. Crude or adjusted (for age and gender) odds ratios (ORs) with respective 95% confidence intervals (CIs) were obtained from logistic regression analyses for the analysis of associations between polymorphisms and neuroblastoma risk. Logistic regression analyses were adopted to obtain haplotype frequencies and distinct haplotypes, with the adjustment for gender and age.^43^, ^44^ The *P* value level of significance was .05. We did the analyses using SAS 9.1 (SAS Institute).

## 3 RESULTS

### 3.1 Associations between *METTL3* SNPs and neuroblastoma susceptibility

The clinical characteristics of the eligible participants (968 cases and 1814 controls) were depicted in Table S1. No significant differences between cases and controls were observed with respect to age (*P* = .536) and gender (*P* = .231). The associations between the four *METTL3* SNPs and neuroblastoma risk were shown in Table 1. The *P* values of HWE for all SNPs were >.05 in the controls, indicating none of them departing from HWE. In the single‐locus analysis, all the selected variants in the *METTL3* gene showed no significant association with neuroblastoma susceptibility. Then, we analysed the combined effect of risk genotypes but still failed to detect any significant association.

**Table 1:** Association between *METTL3* gene polymorphisms and neuroblastoma risk

Genotype	Cases (N = 966)	Controls (N = 1813)	*P* ^a^	Crude OR (95% CI)	*P*	Adjusted OR (95% CI)^b^	*P* ^b^
rs1061026 T>G (HWE = 0.965)							
TT	778 (80.54)	1468 (80.97)		1.00		1.00	
TG	175 (18.12)	327 (18.04)		1.01 (0.82‐1.24)	.925	1.01 (0.82‐1.24)	.932
GG	13 (1.35)	18 (0.99)		1.36 (0.66‐2.80)	.399	1.37 (0.67‐2.80)	.396
Additive			.646	1.04 (0.87‐1.25)	.644	1.04 (0.87‐1.25)	.650
Dominant	188 (19.46)	345 (19.03)	.783	1.03 (0.84‐1.25)	.782	1.03 (0.84‐1.25)	.789
Recessive	953 (98.65)	1795 (99.01)	.399	1.36 (0.66‐2.79)	.401	1.36 (0.67‐2.80)	.397
rs1061027 C>A (HWE = 0.847)							
CC	641 (66.36)	1173 (64.70)		1.00		1.00	
CA	276 (28.57)	569 (31.38)		0.89 (0.75‐1.06)	.177	0.89 (0.75‐1.06)	.179
AA	49 (5.07)	71 (3.92)		1.26 (0.87‐1.84)	.224	1.26 (0.86‐1.84)	.230
Additive			.826	0.99 (0.86‐1.13)	.826	0.98 (0.86‐1.13)	.823
Dominant	325 (33.64)	640 (35.30)	.382	0.93 (0.79‐1.10)	.382	0.93 (0.79‐1.10)	.384
Recessive	917 (94.93)	1742 (96.08)	.153	1.31 (0.90‐1.90)	.154	1.31 (0.90‐1.90)	.159
rs1139130 A>G (HWE = 0.323)							
AA	393 (40.68)	708 (39.05)		1.00		1.00	
AG	437 (45.24)	834 (46.00)		0.94 (0.80‐1.12)	.504	0.95 (0.80‐1.12)	.539
GG	136 (14.08)	271 (14.95)		0.90 (0.71‐1.15)	.410	0.90 (0.71‐1.15)	.408
Additive			.365	0.95 (0.85‐1.06)	.365	0.95 (0.85‐1.06)	.374
Dominant	573 (59.32)	1105 (60.95)	.402	0.93 (0.80‐1.10)	.402	0.94 (0.80‐1.10)	.426
Recessive	830 (85.92)	1542 (85.05)	.537	0.93 (0.75‐1.17)	.537	0.93 (0.74‐1.16)	.520
rs1263801 G>C (HWE = 0.374)							
GG	495 (51.24)	920 (50.74)		1.00		1.00	
GC	382 (39.54)	732 (40.38)		0.97 (0.82‐1.14)	.717	0.97 (0.83‐1.15)	.750
CC	89 (9.21)	161 (8.88)		1.03 (0.78‐1.36)	.849	1.02 (0.77‐1.35)	.893
Additive			.949	1.00 (0.88‐1.12)	.949	1.00 (0.88‐1.12)	.938
Dominant	471 (48.76)	893 (49.26)	.803	0.98 (0.84‐1.15)	.803	0.98 (0.84‐1.15)	.818
Recessive	877 (90.79)	1652 (91.12)	.770	1.04 (0.79‐1.37)	.769	1.03 (0.79‐1.35)	.823
Combine risk genotypes^c^							
0	55 (5.69)	125 (6.89)	.221	1.00		1.00	
1	688 (71.22)	1305 (71.98)		1.20 (0.86‐1.67)	.283	1.20 (0.86‐1.67)	.278
2	202 (20.91)	335 (18.48)		1.37 (0.95‐1.97)	.088	1.37 (0.95‐1.97)	.088
3	20 (2.07)	48 (2.65)		0.95 (0.51‐1.74)	.861	0.94 (0.51‐1.73)	.837
4	1 (0.10)	0 (0.00)		/	/	/	/
0	55 (5.69)	125 (6.89)		1.00		1.00	
1‐4	911 (94.31)	1688 (93.11)	.221	1.23 (0.88‐1.70)	.221	1.23 (0.89‐1.70)	.218

Abbreviations: CI, confidence interval; HWE, Hardy‐Weinberg equilibrium; OR, odds ratio.

^a^ Chi‐square test for genotype distributions between neuroblastoma patients and cancer‐free controls.

^b^ Adjusted for age and gender.

^c^ Risk genotypes were rs1061026 TG/GG, rs1061027 AA, rs1139130 AA/AG, and rs1263801 CC.

### 3.2 Stratification analysis

Table 2 presented the contents of the correlation between *METTL3* gene polymorphisms and neuroblastoma susceptibility in subgroups divided by age, gender, sites of origins, and clinical stages. We only detected that the rs1061027 AA genotype carriers were more likely to have increased neuroblastoma risk among children aged 18 months and under (adjusted OR = 1.87, 95% CI = 1.03‐3.41, *P* = .040) and females (adjusted OR = 1.86, 95% CI = 1.07‐3.24, *P* = .028)) when compared to CC/CA.

**Table 2:** Stratification analysis for the association between risk genotypes and neuroblastoma risk

Variables	rs1061027 (cases/controls)		AOR (95% CI)^a^	*P* ^a^	Risk genotypes (cases/controls)		AOR (95% CI)^a^	*P* ^a^
	CC/CA	AA			0	1‐4		
Age, mo								
≤18	358/711	22/23	**1.87 (1.03‐3.41)**	**.040**	23/55	357/679	1.25 (0.76‐2.07)	.382
>18	559/1031	27/48	1.02 (0.63‐1.65)	.941	32/70	554/1009	1.19 (0.78‐1.84)	.424
Gender								
Females	409/750	27/26	**1.86 (1.07‐3.24)**	**.028**	23/55	413/721	1.37 (0.83‐2.27)	.217
Males	508/992	22/45	0.95 (0.57‐1.61)	.858	32/70	498/967	1.13 (0.73‐1.73)	.592
Sites of origin								
Adrenal gland	244/1742	16/71	1.60 (0.91‐2.80)	.100	12/125	248/1688	1.52 (0.83‐2.80)	.174
Retroperitoneal	324/1742	19/71	1.43 (0.85‐2.40)	.180	23/125	320/1688	1.03 (0.65‐1.63)	.900
Mediastinum	224/1742	9/71	1.02 (0.50‐2.07)	.957	14/125	219/1688	1.17 (0.66‐2.08)	.585
Others	113/1742	5/71	1.11 (0.44‐2.81)	.822	5/125	113/1688	1.68 (0.67‐4.19)	.266
INSS stages								
I + II +4s	480/1742	27/71	1.40 (0.89‐2.20)	.150	24/125	483/1688	1.49 (0.95‐2.34)	.080
III + IV	403/1742	19/71	1.12 (0.67‐1.89)	.665	31/125	391/1688	0.93 (0.62‐1.40)	.732

Abbreviations: AOR, adjusted odds ratio; CI, confidence interval.

^a^ Adjusted for age and gender, omitting the corresponding stratification factor.

The results were in bold if the 95% CIs excluded 1 or *P* values less than 0.05.

### 3.3 Haplotype analysis

We further examined whether the haplotypes of the four *METTL3* gene SNPs are correlated to neuroblastoma risk in an order of rs1061026, rs1061027, rs1139130 and rs1263801. As shown in Table 3, the TCGG haplotype was defined as the reference group. We failed to detect a significant relationship between neuroblastoma risk and subjects with all the haplotypes.

**Table 3:** Association of inferred haplotypes of *METTL3* gene based on observed genotypes with neuroblastoma susceptibility

Haplotypes^a^	Cases (N = 1932)	Controls (N = 3626)	Crude OR (95% CI)	*P*	Adjusted OR^b^ (95% CI)	*P* ^b^
TCGG	199 (10.30)	416 (11.47)	1.00		1.00	
TCGC	50 (2.59)	78 (2.15)	1.34 (0.90‐1.99)	.145	1.34 (0.90‐1.98)	.148
TCAG	1129 (58.44)	2082 (57.42)	1.13 (0.94‐1.36)	.181	1.13 (0.94‐1.36)	.185
TCAC	22 (1.14)	56 (1.54)	0.82 (0.49‐1.38)	.459	0.81 (0.48‐1.37)	.433
TAGG	30 (1.55)	62 (1.71)	1.01 (0.63‐1.61)	.962	1.02 (0.64‐1.62)	.949
TAGC	293 (15.17)	569 (15.69)	1.08 (0.86‐1.34)	.512	1.08 (0.86‐1.34)	.515
TAAG	8 (0.41)	0 (0.00)	/	/	/	/
TAAC	0 (0.00)	0 (0.00)	/	/	/	/
GCGG	1 (0.05)	4 (0.11)	0.52 (0.06‐4.71)	.563	0.54 (0.06‐4.86)	.582
GCGC	94 (4.87)	167 (4.61)	1.18 (0.87‐1.60)	.294	1.18 (0.87‐1.60)	.290
GCAG	5 (0.26)	7 (0.19)	1.49 (0.47‐4.76)	.498	1.50 (0.47‐4.79)	.494
GCAC	58 (3.00)	105 (2.90)	1.16 (0.80‐1.66)	.437	1.15 (0.80‐1.66)	.443
GAGG	0 (0.00)	1 (0.03)	/	/	/	/
GAGC	42 (2.17)	79 (2.18)	1.11 (0.74‐1.68)	.614	1.10 (0.73‐1.66)	.643
GAAG	0 (0.00)	0 (0.00)	/	/	/	/
GAAC	1 (0.05)	0 (0.00)	/	/	/	/

Abbreviations: CI, confidence interval; OR, odds ratio.

^a^ The haplotype order was rs1061026, rs1061027, rs1139130 and rs1263801.

^b^ Obtained in logistic regression models with adjustment for age and gender.

## 4 DISCUSSION

Understanding the genetic backgrounds of neuroblastoma will help to improve prevention strategy and precision medicine. In this large Chinese case‐control study, we investigated whether genetic variations in the *METTL3* gene contribute to neuroblastoma risk. We unveiled a weak association between the *METTL3* gene SNPs and an elevated neuroblastoma risk in a Chinese population.

METTL3 is a catalytic enzyme of m^6^A methyltransferase systems. It usually forms a stable heterodimeric complex with METTL14. The m^6^A RNA modification catalysed by METTL3 is a post‐transcriptional mechanisms regulating gene expression, which is involved in pre‐mRNA splicing,^45^ mRNA decay,^46^ mRNA translation^47^ and miRNA processing.^48^ Growing evidence has suggested the implication of METTL3 in diverse human cancers. METTL3 was up‐regulated in clear cell renal cell carcinoma in comparison with normal samples.^49^ Patients with METTL3 deletion had poor overall survival and disease‐free survival. Li et al^50^ showed that METTL3 expression was higher in colorectal carcinoma metastatic tissues and was associated with a poor prognosis. Functional experiments revealed that METTL3 acts as an oncogene via a m^6^A‐IGF2BP2‐dependent mechanism. In contrast, some studies have shown that METTL3 might be a tumour suppressor. Deng et al^51^ observed that high expression of METTL3 was strongly correlated with better prognosis, indicating its tumour‐suppressive role in colorectal cancer. Zhao et al^52^ found that knockdown of METTL3 significantly promoted the proliferation of bladder cancer cell line and knockin of wild‐type METTL3 could restore their normal growth rate. In view of METTL3’s specific role in different cancers, further studies are still needed to clarify the exact roles of METTL3 in specific type of cancers.

*METTL3* gene resides in chromosome 14q11.2. There is only one research regarding the impact of *METTL3* gene SNPs on cancer risk. Meng et al conducted the first case‐control study on the association between m^6^A modification gene SNPs and cancer risk. Their study includes two stages, discovery stage with 1150 colorectal cancer cases and 1342 controls, and validation stage with 932 colorectal cancer cases and 966 controls. Among the 240 SNPs in 20 m^6^A modification‐related genes, only *SND1* gene rs118049207 predisposed to colorectal cancer in the Chinese population. All the ten investigated *METTL3* SNPs rs3752411, rs1263793, rs2242526, rs1268403, rs113058369, rs1263797, rs1263796, rs11851342, rs1263800 and rs10450908 were not associated with colorectal cancer risk.^53^ In the present study, we performed this first epidemiology study to determine the correlation of *METTL3* gene polymorphisms and neuroblastoma risk in a Chinese population. Our results showed that rs1061027 A allele was likely to increase neuroblastoma risk in children ≤ 18 months of age and girls.

Our study has several potential limitations. Though our study used by far the largest Chinese cohort to explore neuroblastoma risk‐associated SNPs, stratification analysis suffers from reduced statistical power due to the rarity of the samples. Second, we had no access to other environmental factors, which could have biased cancer risk assessment due to the lack of adequate adjustment for these covariates in the risk evaluation model. Finally, because all selected subjects were from Chinese populations, the results may not be extrapolated to other races. Therefore, replication studies should be carried out in additional ethnicities.

Collectively, we elucidated the predisposing role of *METTL3* gene SNPs to neuroblastoma risk. Replication of the findings in a larger study population is warranted.

## CONFLICT OF INTEREST

The authors confirm that there are no conflicts of interest.

## AUTHOR CONTRIBUTION

**Jun Bian:** Conceptualization (equal); Investigation (equal); Resources (equal); Writing‐original draft (equal); Writing‐review & editing (supporting). **Zhenjian Zhuo:** Investigation (equal); Writing‐original draft (equal); Writing‐review & editing (supporting). **Jinhong Zhu:** Investigation (supporting); Writing‐review & editing (equal). **Zhonghua Yang:** Investigation (supporting); Resources (equal); Writing‐review & editing (supporting). **Zhang Jiao:** Investigation (supporting); Resources (equal); Writing‐review & editing (supporting). **Yong Li:** Investigation (supporting); Resources (equal); Writing‐review & editing (supporting). **Jiwen Cheng:** Investigation (supporting); Resources (equal); Writing‐review & editing (supporting). **Haixia Zhou:** Investigation (supporting); Resources (equal); Writing‐review & editing (supporting). **Suhong Li:** Investigation (supporting); Resources (equal); Writing‐review & editing (supporting). **Li Li:** Investigation (supporting); Resources (equal); Writing‐review & editing (supporting). **Jing He:** Conceptualization (lead); Data curation (lead); Formal analysis (lead); Funding acquisition (lead); Investigation (equal); Methodology (lead); Project administration (lead); Resources (equal); Software (lead); Supervision (lead); Validation (equal); Visualization (equal); Writing‐original draft (supporting); Writing‐review & editing (lead). **Yanfei Liu:** Investigation (supporting); Resources (equal); Writing‐review & editing (supporting).

## Supporting information

Supplementary Material

## Data Availability

All the data were available upon request.
