# Supplementary material for: Association between METTL3 gene polymorphisms and neuroblastoma susceptibility: A nine‐centre case‐control study
Source: J Cell Mol Med. 2020 Jul 2;24(16):9280–6. doi: 10.1111/jcmm.15576 (PMC7417682; doi:10.1111/jcmm.15576)
Supplement: Supplementary file 1 — Supplementary Material [file JCMM-24-9280-s001.doc]

| **Supplemental Table 1**. Frequency distribution of selected characteristics in neuroblastoma cases and cancer-free controls | | | | | |
| --- | --- | --- | --- | --- | --- |
| Variables | Combined subjects (9 Centers) | | | | |
|  | Cases (N=968) | | Controls (N=1814) | | *P* ^a^ |
|  | No. | % | No. | % |  |
| Age range, month | 0.00-176.00 | | 0.004-156.00 | | 0.536 |
| Mean ± SD | 32.85±28.22 | | 31.67±26.40 | |  |
| ≤18 | 380 | 39.26 | 734 | 40.46 |  |
| >18 | 588 | 60.74 | 1080 | 59.54 |  |
| Gender |  |  |  |  | 0.231 |
| Female | 437 | 45.14 | 776 | 42.78 |  |
| Male | 531 | 54.86 | 1038 | 57.22 |  |
| INSS stages |  |  |  |  |  |
| I | 344 | 35.54 | / | / |  |
| II | 164 | 16.94 | / | / |  |
| III | 169 | 17.46 | / | / |  |
| IV | 253 | 26.14 | / | / |  |
| 4s | 20 | 2.07 | / | / |  |
| NA | 18 | 1.86 | / | / |  |
| Sites of origin |  |  |  |  |  |
| Adrenal gland | 260 | 26.86 | / | / |  |
| Retroperitoneal region | 344 | 35.54 | / | / |  |
| Mediastinum | 234 | 24.17 | / | / |  |
| Other region | 118 | 12.19 | / | / |  |
| NA | 12 | 1.24 | / | / |  |
| SD, standard deviation; NA, not available.  ^a^ Two-sided χ^2^ test for distributions between neuroblastoma cases and cancer-free controls. | | | | | |


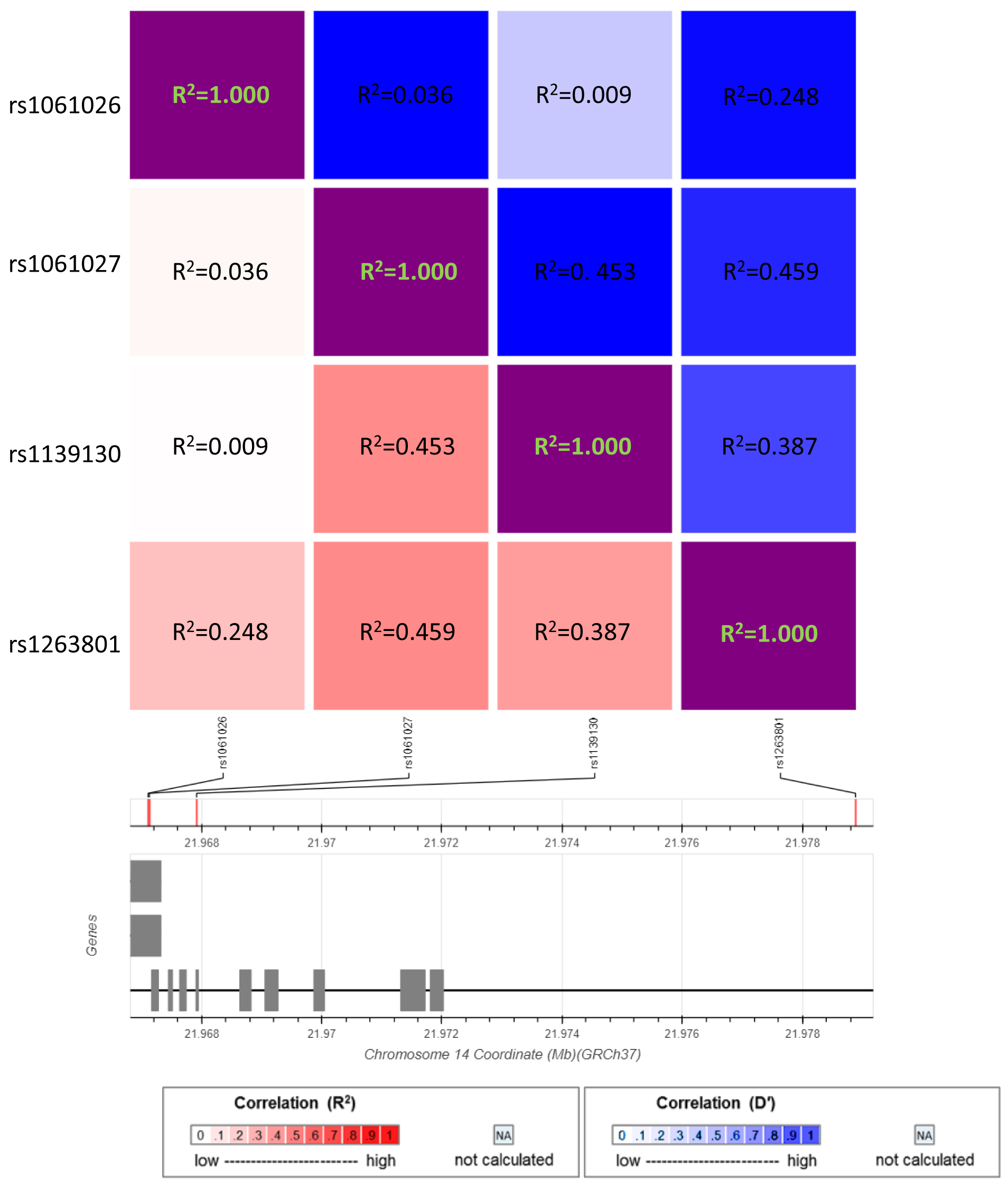


**Supplemental Figure 1.** Linkage disequilibrium (LD) analysis for the four selected SNPs in *METTL3* gene in Chinese Han population consisting of CHB (Han Chinese in Beijing, China) and CHS (Southern Han Chinese) subjects. LD as R^2^ for SNP pairs is shown inside the squares.
